# A Method for Isolating and Culturing Skin Cells: Application to Endothelial Cells, Fibroblasts, Keratinocytes, and Melanocytes From Punch Biopsies in Systemic Sclerosis Skin

**DOI:** 10.3389/fimmu.2020.566607

**Published:** 2020-10-07

**Authors:** Pauline Henrot, Paôline Laurent, Emeline Levionnois, Damien Leleu, Catherine Pain, Marie-Elise Truchetet, Muriel Cario

**Affiliations:** ^1^Univ. Bordeaux, Inserm, BMGIC, UMR1035, Bordeaux, France; ^2^Department of Rheumatology, National Reference Center for Systemic Autoimmune Rare Diseases, Hopital Pellegrin, Bordeaux, France; ^3^Univ. Bordeaux, CNRS, Immunoconcept, UMR 5164, Bordeaux, France; ^4^Department of Dermatology and Pediatric Dermatology, National Center for Rare Skin Disorders, Hôpital Saint André, Bordeaux, France; ^5^AquiDerm, Univ. Bordeaux, Bordeaux, France

**Keywords:** scleroderma, cell culture, human microvascular dermal endothelial cells, dermis, epidermis

## Abstract

Systemic Sclerosis (SSc) is a complex auto-immune connective tissue disease combining inflammatory, vasculopathic and fibrotic manifestations. Skin effectively recapitulates the main pathogenic processes and therefore is a good organ to decipher the disease pathophysiology, which remains unclear. However, culturing primary skin cells is SSc can be a major issue due to small sample size combined to skin fibrosis. Here, we present a protocol allowing to isolate and culture the four main types of skin cells: dermal cells (microvascular dermal endothelial cells—HDMECs—and fibroblasts) and epidermal cells (keratinocytes and melanocytes), from a single 4 mm-punch biopsy, at a low cost. The present protocol has been optimized to fit SSc skin cells particularities. Such technique allows to culture primary cells, crucial to study the disease pathophysiology, as well as to isolate cells in order to perform immediate molecular biology experiments such as single-cell transcriptomic. Cells grown from biopsies are also suitable for various types of experiments such as immunocytochemistry, Western blot, RT-qPCR or functional *in vitro* assays (angiogenesis, migration, etc.). Ultimately, they can be used for experimental 3D cell culture models such as reconstructed skin.

## Introduction

Systemic Sclerosis (SSc) is a rare auto-immune connective tissue disease combining autoimmune features, widespread vasculopathy, and systemic fibrosis (1). Despite recent therapeutic advances, the morbidity and mortality remain high, with a standardized mortality ratio up to 7.2-fold according to studies (2). Skin manifestations, which give the original name to the disease (scleroderma), are considered as diagnostic, subclassification, severity, and prognostic markers (3). SSc skin effectively recapitulates the main pathogenic processes of the disease, while being the most accessible organ to biopsy. Therefore, SSc skin cells are extremely helpful to decipher the disease pathophysiology, which remains unclear. However, due to ethical issues, only small biopsies can be performed in patients, contrary to healthy skin, which can usually be obtained in larger samples; moreover, SSc skin features (notably fibrosis and vasculopathy) further add to the difficulty of isolating and culturing cells.

Here, we propose a technique allowing the isolation and primary cell culture of the four main types of skin cells: epidermal cells (keratinocytes and melanocytes) and dermal cells (fibroblasts and human dermal microvascular endothelial cells or HDMECs). This protocol, which is applicable to every skin disease, has been applied to fit the particularities of SSc skin cells. Briefly, the protocol consists in splitting the epidermis from the dermis after an incubation with dispase, and separately seeding epidermal cells (keratinocytes and melanocytes) and dermal cells (HDMECs and fibroblasts). Endothelial cells are obtained by a mechanical extraction combined with immuno-magnetic cell sorting, thus avoiding contamination by fibroblasts, which is a major issue. Fibroblasts are further obtained by digesting the dermis in collagenase. Keratinocytes and melanocytes are purifiedafter a few days of primary culture by means of a “differential trypsinization” technique. All cells can be passaged rapidly after the initial seeding and used for cell culture experiment as well as molecular and biochemical analysis (immunocytochemistry, Western blotting, qPCR). Such technique also allows performing immediate molecular biology experiments such as single-cell transcriptomic. Finally, cultured cells can be implemented into skin equivalents models, such as reconstructed skin, in order to reproduce the complex cellular interactions within the skin microenvironment.

## Materials and Equipment

### Material for Tissue Digestion and Primary Cells Seeding

Day 0 (D0): sample arrival, tissue digestion

Equipment: Ethanol 70°, simple clamp

Reagents:

- Hank's Balanced Salt Solution (HBSS) with calcium and magnesium (optional: with phenol red): e.g., Gibco
- Dispase II solution: 25 UI/mL in HBSS, e.g., 4942078001, Roche

Day 1 (D1): primary cells seeding

Equipment:

- Ethanol 70°
- curved clamp
- gripped clamp
- sterile surgical scalpel blade (we recommend size 24, which makes cutting easier) and blade holder
- Falcon 70 μm cell strainer: e.g., 352350, Corning

Reagents:

- HBSS
- Fetal Calf Serum (FCS): sterile and heat inactivated at 56° during 30 min, e.g., CVFSVF00-01, Eurobio
- Trypsin-EDTA (TE): Trypsin 0.25%, e.g., T9201, Sigma—Ethylenediaminetetraacetic acid (EDTA) 0.1%, e.g., E4884, Sigma in HBSS

Q-medium:

Reagents:

- Iscove's Modified Dulbecco's Media (IMDM), optional: with phenol red, e.g., Gibco
- Fetal Calf Serum (FCS), sterile and heat inactivated at 56° during 30 min, e.g., CVFSVF00-01, Eurobio
- Penicilline-Streptomycine (PS), e.g., CABPES01-0U, Eurobio

Q-medium composition: IMDM + 10% FCS + 1% PS

- Collagenase solution: 40 UI/mL in HBSS, e.g., C-9891, Sigma
- Cell culture media: (add 1% PS for each)
  - Melanocytes: Melanocyte Growth Medium (MGM), C-24010, Promocell
  - Endothelial cells: Endothelial Cell Growth Medium (MV2), C-22022, Promocell
  - Fibroblasts: Dulbecco's Modified Eagle's medium, e.g., Gibco + 10% FCS

All reagents prepared from powders must be sterile filtered through a 0.22 μm-filter.

Further cell culture and amplification:

- Keratinocyte Growth Medium 2 (KGM2), Promocell
- For cell passaging:

TE 10%: 0.025% Trypsin-0.01 % EDTA diluted 1/10^e^ in HBSS

FCS 10%: FCS diluted 1/10^e^ in HBSS

*Nota bene*: for cell culture flasks, better use vented caps.

All human material was obtained thanks to the biomedical research cohort VISS (Vasculopathy and Inflammation in Systemic Sclerosis) approved by the institutional ethical committee (CPP, 2012 A00081-42, Aquitaine). All patients provided written and informed consent before the biopsy.

### Material for Immunomagnetic HDMECs Sorting

For CD31^+^ cells sorting:

- Manual separator, e.g., QuadroMACS^TM^ Separator (MACS)
- Immunomagnetic sorting anti-CD31 microbeads: e.g., 130-091-935, Miltenyi.
- LS columns (Miltenyi)
- TE 10%
- FCS 10%
- HBSS
- Sorting buffer (optional): Phosphate-Buffered Saline (PBS) + 2 mM EDTA + 0.5% Bovine Serum Albumin (BSA)

(NB: for a low number of cells, MS columns are usually recommended. However, we found that LS columns were more efficient for HDMECs sorting, possibly due to a quicker flow of cell suspension through the column, exposing cells to a shorter duration of stressful conditions).

Additional material for CD45^−^ cells sorting:

- LD columns (Miltenyi).
- anti-CD45 microbeads (e.g., 130-045-801, Miltenyi).

### Material for HDMECs Phenotyping (by Fluorescence-Activated Cell Sorting)

- anti-CD31 fluorochrome-conjugated antibody: e.g., APC-conjugated anti-CD31 antibody, clone AC128, Miltenyi
- anti-CD45 fluorochrome-conjugated antibody: e.g., FITC-conjugated anti-CD45 antibody, clone 5B1, Miltenyi
- FACS buffer: e.g., PBS + 2 mM EDTA + 0.5% BSA
- Flow cytometer: e.g., Canto I, BD.

## Methods

**Figure 1:**
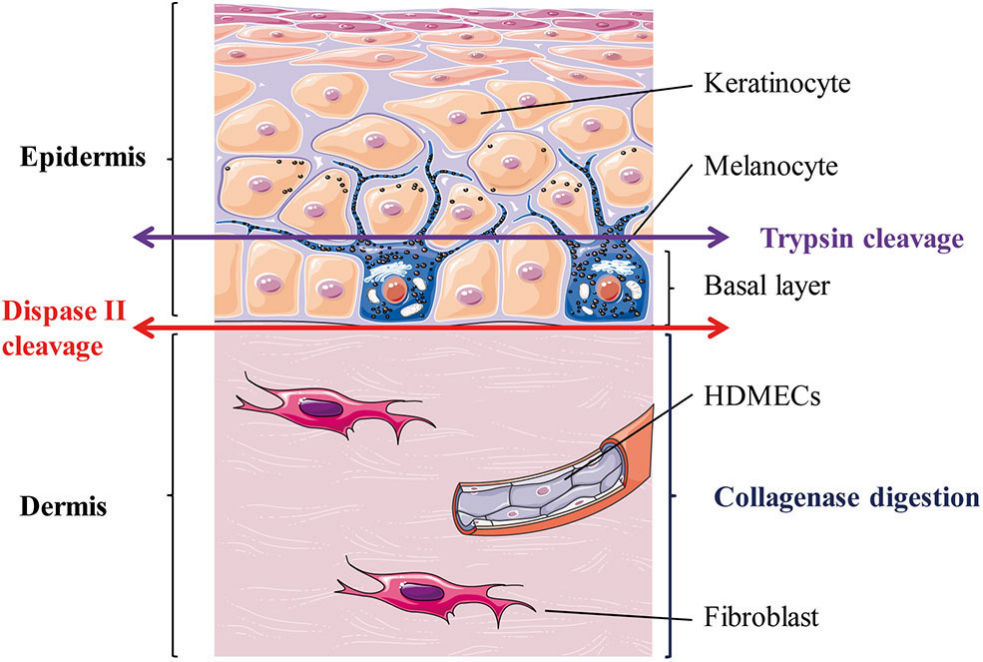
Skin layers and general principle of the enzymatic dissociation used in the protocol. HDMECs, human dermal microvascular endothelial cells. The epithelial layer is the epidermis and is composed mostly of keratinocytes (90%) and melanocytes (5%). Melanocytes sit at the epidermal basal layer, which also contains keratinocytes stem cells, which differentiate as they reach upper layers. The connective tissue layer is the dermis and is composed mostly of extra-cellular matrix secreted by fibroblasts and is perfused with blood vessels, whose internal layer is composed of dermal microvascular endothelial cells (HDMECs).

**Figure 2:**
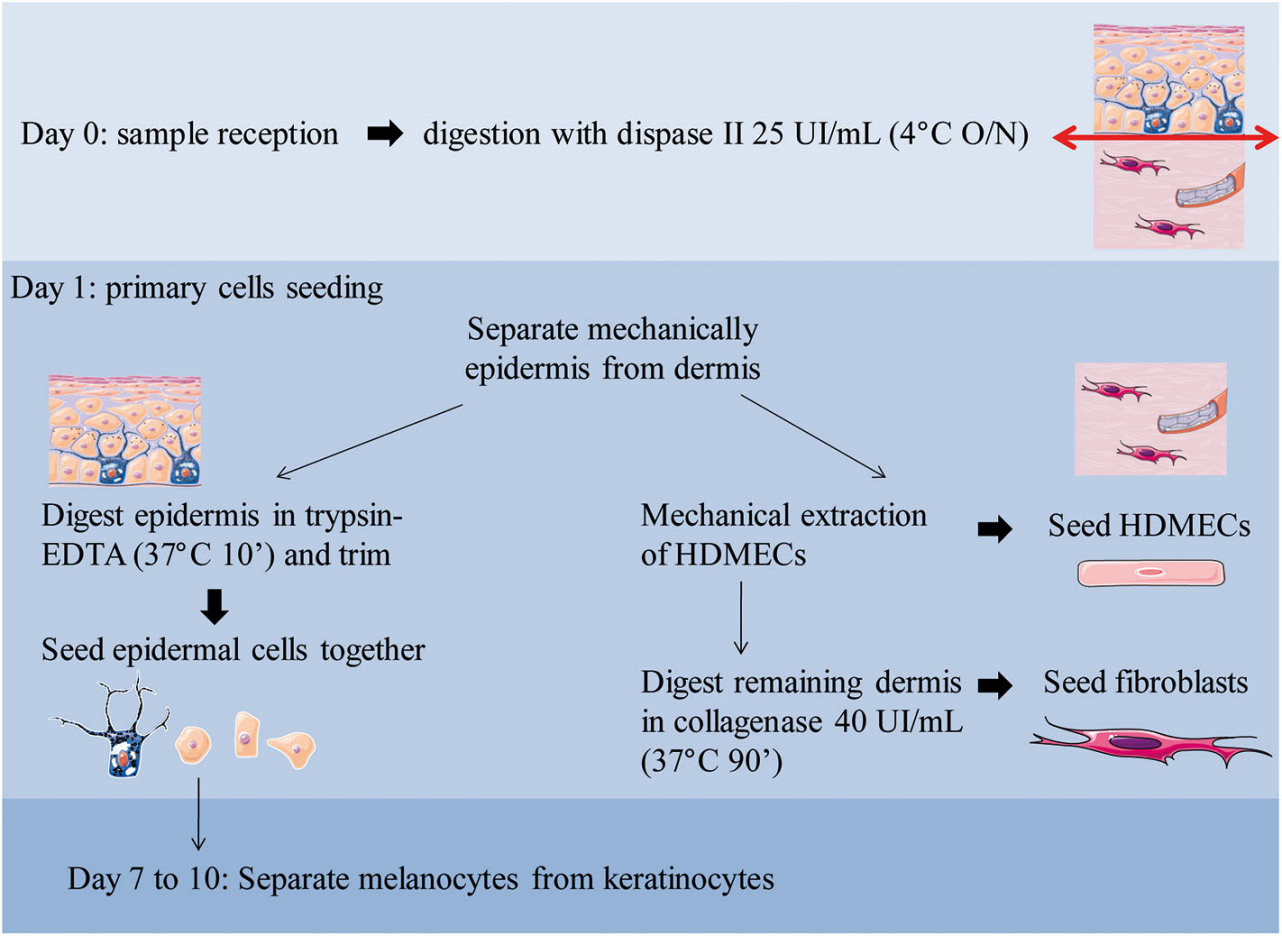
Overview of the workflow with daily steps. O/N, overnight.

**Figure 3:**
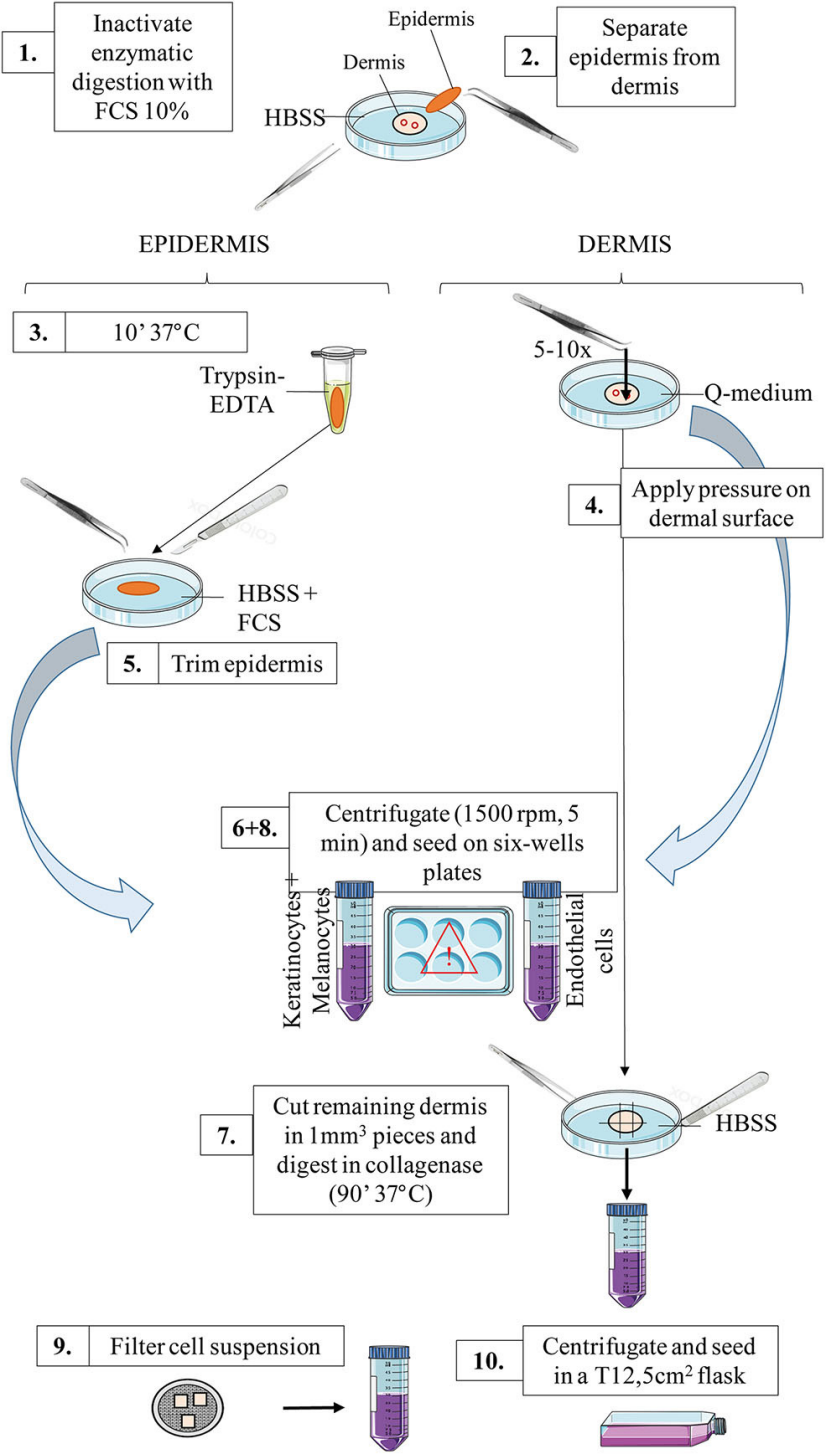
Schematic representation of the workflow for day 1: primary cells seeding. For a detailed explanation of each step, cf section Day 1: Primary Cells Seeding.

Figure 1 outlines skin cells *in situ* and protocol principle. Steps of the whole protocol are presented Figure 2 (for whole protocol outlining) and Figure 3 (for day 1: cell seeding).

### Day 0: Sample Arrival, Tissue Digestion

The sample (ideally a 4-mm biopsy punch minimum, but smaller punch such as 3-mm should do as well) should have been conserved in a sterile pad soaked with saline solution (alternatively, submerged in sterile saline solution).

Dip the sample rapidly in ethanol (to avoid future contamination), then rinse thoroughly in HBSS. Incubate the whole sample in dispase solution (25 UI/mL), overnight à 4°C (the sample can be put in a 1.5 mL tube containing 1 mL dispase) (maximal incubation time: 15 h).

Alternatively, if necessary: incubate the sample in dispase for 90 min at 37°C.

### Day 1: Primary Cells Seeding

Prepare sterile material and reagents according to the procedure described in section Material for Tissue Digestion and Primary Cells Seeding. For clamps: dip in ethanol before and after each use to ensure sterility, then dip in sterile HBSS before using again.

Cell seeding: (protocol illustration: Figures 2,3):

1. Transfer the tube content into a Petri dish. Inactivate the enzymatic digestion (dispase) by adding the same volume of FCS 10% in HBSS.
2. Mechanically separate the epidermis from the dermis. The epidermis should be a very thin sheet, easily peeled off the dermal part.
  Tip: for an easy separation, hold the dermal part with the gripper clamp whilst grasping and peeling the epidermis off (in one piece) with the curved clamp (Supplementary Video 1).
3. (Epidermal layer) Transfer the epidermal sheet into a 1.5 mL tube containing 1 mL Trypsin-EDTA and incubate at 37°C for 10 min (incubator or water bath).
4. (Dermal layer) Meanwhile: in a Petri dish filled with Q-medium, dip the dermal part and mechanically extract HDMECs.
  To this end, apply gently but firmly the curved clamp on the dermal surface (cf Supplementary Video 2). Best results are obtained if you look for and press against microvessels on the deep dermal part, and rinse the dermal surface with Q-medium afterwards.
  **This step is highly critical**, as too much pressure will result in fibroblasts contamination, while too few pressure will not extract enough HDMECs. Several tests with healthy skin are advised in order for experimentators to fine-tune their gesture before starting with patient's samples.
  Collect the Q-medium in a 50 mL tube and set aside the dermal piece in a Petri dish filled with HBSS.
5. (Epidermal layer) Shake vigorously the tube containing the epidermal sheet after the 10 min incubation of step 3 and transfer the tube content in a Petri dish filled with HBSS. Inactivate the enzymatic digestion of the epidermis by adding the same volume of FCS. Then, dissociate the epidermal cells by trimming the epidermis in a Petri dish filled with HBSS.
  Tip: for an easy cutting, hold the epidermal sheet with the curved clamp while trimming with the scalpel blade until maximum shredding. This step is easier in the lid of the Petri dish, as the edges are lower.
  Collect the epidermal pieces as well as the supernatant in a 50 mL tube.
  If necessary, adjust the volume of the two tubes with HBSS before centrifugation.
6. Centrifugate epidermal and endothelial cells at the appropriate speed for cells [e.g., 5 min, 1,200 rotations per minute (rpm)].
7. (Dermal layer) Meanwhile, cut the remaining dermal part into 1 mm^3^ pieces using the scalpel blade. Transfer the pieces in a 50 mL tube containing 5 mL of collagenase solution. Incubate for 90 min at 37°C under mechanical shaking. Alternatively, if no mechanical shaker is available, incubate the tube at 37°C and manually shake it every 10–15 min.
8. After the centrifugation of step 6, do not discard the supernatant!
  For HDMECs: Collect the supernatant and seed into a 6-wells plate: two wells filled with 1 mL of endothelial cells culture medium. Resuspend the cell pellet with 2 mL of culture medium and seed in a third well (named “pellet”). Seeding three separate wells maximizes the chances to obtain a pure HDMECs culture afterwards.
  For epidermal cells: Collect the supernatant and seed into 2–4 wells (depending on the initial volume) filled with 1 mL of melanocytes culture medium. Resuspend the cell pellet with 4 mL of culture medium and seed in two to four separate wells.
9. After the 90 min incubation of remaining dermal cells (fibroblasts) described in step 7, inactive the enzymatic digestion by adding the same amount of FCS 10% directly into the tube. Then, filter the cell suspension through a 70 μm-cell strainer. To this end, gently press the skin pieces above the filter using the plunger from a 10 mL-syringe. Rinse two to three times with HBSS.
10. Centrifugate the filtered suspension 5 min at 1,200 rpm. Resuspend the cell pellet with 3 mL of fibroblasts culture medium and seed into a 12.5 cm^2^-flask.

### Day 2 (to 4 if Necessary): First Cell Medium Renewal

- For the epidermal cells (keratinocytes/melanocytes) plate: collect the supernatant of all wells and rinse with HBSS; centrifugate the collected supernatant and resuspend the pellet with fresh medium before distributing it equally inside all wells (new wells in case of many adhered cells). This step is to ensure optimal adhesion of initially seeded cells.
- For the endothelial cells plate: add 1 mL of fresh medium to the well named “pellet.” Collect the supernatant of the two other wells, centrifuge and resuspend the pellet with fresh medium before distributing it equally between all others wells.
- For the fibroblast flask (optional): if a lot of floating debris are present, collect the supernatant, rinse the flask with HBSS; centrifuge the collected medium, discard the supernatant of the centrifuged tube and resuspend the pellet with fresh medium before adding it again into the flask.

### Fibroblasts Amplification

When fibroblasts reach 70–80% confluence, detach cells using the following procedure:

- Aspirate and discard the supernatant, rinse the flask with HBSS, add 0.5 mL of TE 10%
- Incubate at 37°C during 3–5 min, regularly check under the microscope if cells are detached; gently tap the flask if necessary
- When cells are detached, block TE action with FCS 10%. Collect the cell suspension, centrifuge 5 min at 1,500 rpm. Discard the supernatant, resuspend the cell pellet into 8 mL of fibroblasts culture medium and seed into 2 T25 cm^2^ flasks (or 6-wells plates depending on further experiments).

Alternatively, freeze the cells if necessary. A minimum of 100 000–200 000 cells per vial is necessary to ensure that cells will grow back after thawing (concentration minimum: 1 million cells/mL of freezing medium). An example of freezing medium is culture medium supplemented with FCS + 10% dimethylsulfoxyde (DMSO).

### HDMECs Amplification ± Purification

HDMECs primoculture gives rise to several HDMECs islets which will grow independently in a concentric manner. When the islets have sufficiently grown, even if the well is not confluent (Supplementary Figure 1), each well can be passaged into a 25 cm^2^-flask according to the procedure described in section Fibroblasts Amplification.

Important point: always check the phenotype of HDMECs culture by labeling cells with an anti-CD31 fluorochrome-conjugated antibody and performing fluorescence-activated cell sorting. A minimum of 10 000 labeled cells is needed for this experiment (see protocol in Supplementary Material). A purity of >90% CD31^+^-cells is acceptable. As immune cells can also express CD31 (4), it is best to perform a simultaneous labeling with an anti-CD45 fluorochrome-conjugated antibody to rule out the presence of double-positive cells (which would be immune cells).

**Figure 4:**
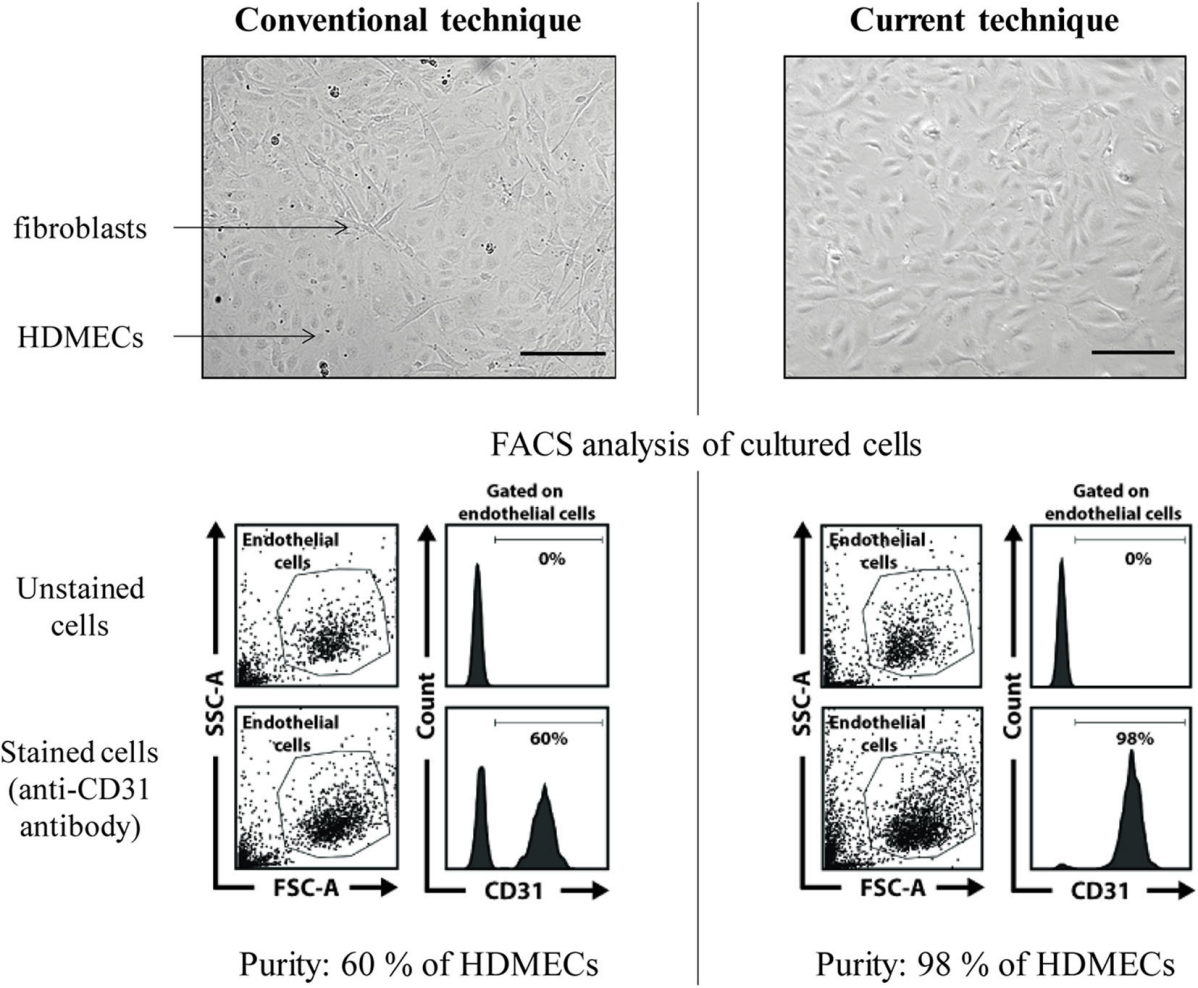
HDMECs culture: efficacy of the proposed protocol (mechanical extraction) vs. conventional technique (dermal digestion with collagenase). FACS, fluorescence-activated cell sorting. Scale bar, 100 μm. In the conventional technique example picture, HDMECs islets (plain arrow) are surrounded by fibroblasts (dotted arrow). FACS analysis of cultured cells shows a proportion of 60% of HDMECs, identified thanks to CD31 surface marker expression. In the proposed technique, pure HDMECs culture can be seen, as confirmed by FACS analysis showing 98% of CD31+ cells in the culture plate.

This mechanical extraction of HDMECs is very efficient and fibroblasts contamination is rare (Figure 4). However, if fibroblasts contamination occurs, which is easy to spot by observing cell culture, as HDMECs islets and fibroblasts differ in shape, two additional methods are possible:

- “differential trypsinization” technique:

This technique is usually efficient within 5–7 days of primary cell culture, before fibroblasts grow too much and HDMECs adhesion is too strong. When several islets of HDMECs appear, try to detach selectively HDMECs by applying TE 10% and checking under the microscope that fibroblasts remain attached. Collect the cell suspension and seed with a 1:2 split ratio.

- immunomagnetic cell sorting:

This technique can be used in case of failure of differential trypsinization or in later stages of cell culture. If the culture is still not pure afterwards, perform one immunomagnetic cell sorting with anti-CD31 microbeads (see protocol in Supplementary Material). The CD31 negative cells are fibroblasts and can be cultured in the appropriate medium after cell sorting.

Another possible contamination would be the presence of immune cells, such as lymphocytes or dendritic cells. To avoid this, another immunomagnetic cell sorting can also be performed at that stage in order to deplete CD45-positive cells (immune cells) (see protocol in Supplementary Material).

If necessary, HDMECs can be freezed until further use. A minimum of 100 000–200 000 cells per vial is necessary to ensure that cells will grow back after thawing (concentration minimum: 1 million cells/mL of freezing medium). An example of freezing medium is culture medium + 10% FCS + 10% DMSO.

### Keratinocytes and Melanocytes Separation

**Figure 5:**
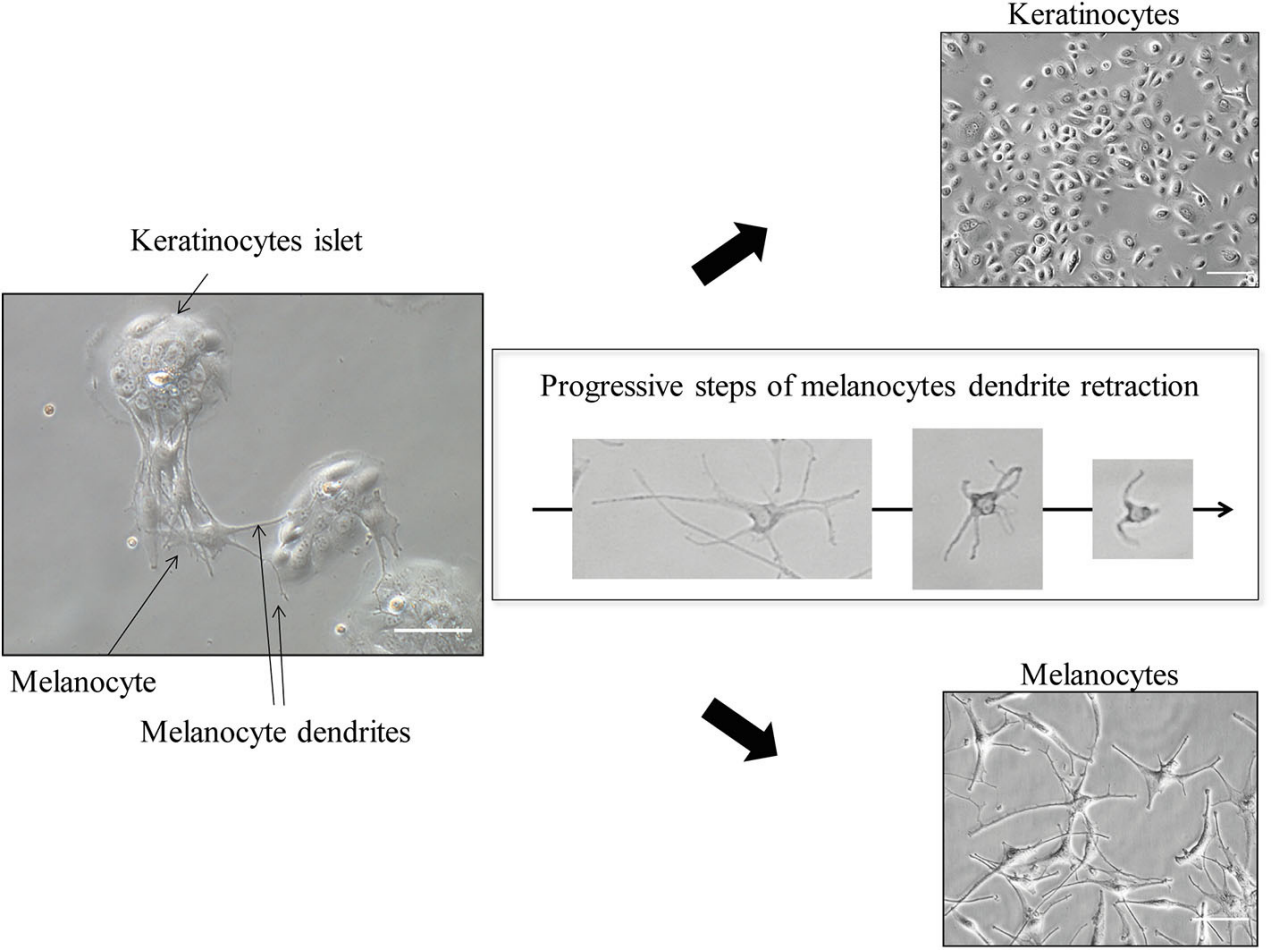
Keratinocytes and Melanocytes separation (≪ differential trypsinization ≫). Scale bar for co-culture and for keratinocytes alone (upper right), 100 μm; for melanocytes alone (lower right), 50 μm. After trypsin application, melanocytes dendrites progressively retract within a few minutes, allowing easy detachment and leaving pure keratinocytes culture in the initial dish.

When epidermal cells reach 60–70% confluence (this should be about 7–10 days from the initial seeding), separate melanocytes from keratinocytes (Figure 5). This is facilitated by the weaker adhesion system of melanocytes compared to keratinocytes; therefore, melanocytes detach before keratinocytes when challenged with trypsin.

To this end, apply TE 10% (e.g., 500 μL per well) in the wells and check under the microscope for dendrites retraction (the melanocytes will become round). After 1 or 2 min, collect the detached cells (which are the melanocytes)—do not wait too much, as you will get keratinocytes coming along—and block TE 10% with FCS 10% poured directly into the collection tube. Centrifugate this collection tube (5 min, 1,200 rpm).

Meanwhile, if necessary, apply again TE 10% on the initial well to ensure getting rid of every melanocyte (check under the microscope). Then, rinse the well (which contains only keratinocytes) with 10% FCS. Rinse again with HBSS, as prolonged FCS exposure leads to keratinocytes differentiation. Carefully pour fresh keratinocytes culture medium on the well and put the plate back into the incubator.

After centrifugation of the melanocytes tube, resuspend the cell pellet with Melanocyte Growth Medium, count the cells and seed into a 12-wells plate at the concentration of minimum 50 000 cells per well. Alternatively, seed them in order to perform immunocytochemistry experiment, for example in lab-tek^TM^ chamber slides.

Tip: melanocytes do not grow very well in very large culture flasks; best results are obtained when they are cultured in small spaces such as 12 or 24-wells plates.

From our personal experience, melanocytes do not grow back well after freezing.

### Keratinocytes Culture and Amplification

After a few days, the keratinocytes will be ready to be expanded into a bigger flask (usually one T25 cm^2^-flask starting from the whole 6-wells-plate), according to the procedure described in section Fibroblasts Amplification. For further experiments, use keratinocytes of passage 2 or fewer. Further passages might compromise the results as it favors cell differentiation.

If necessary, keratinocytes can be freezed until further use, although it should be avoided if possible, or done at the earliest passage. A minimum of 100 000–200 000 cells per vial is necessary to ensure that cells will grow back after thawing (concentration minimum: 1 million cells/mL of freezing medium). An example of freezing medium is culture medium + 10% FCS + 10% DMSO.

## Results

### Cell Purification and Culture

**Figure 6:**
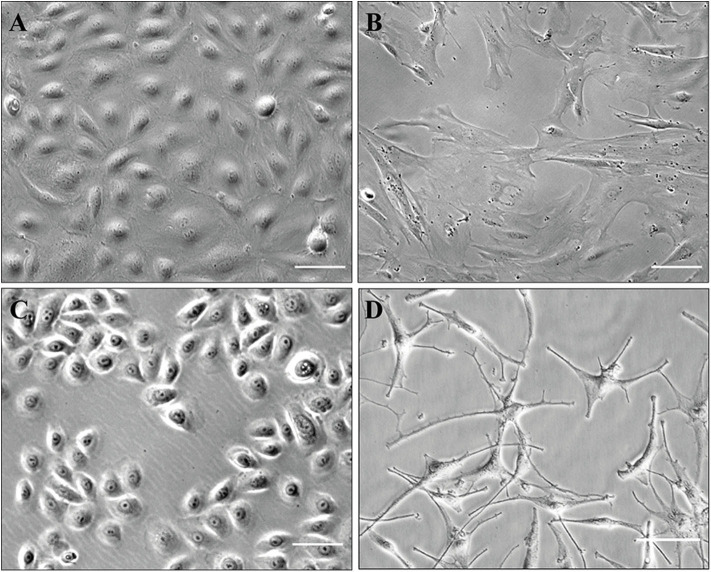
Illustration of cultured cells (brightfield phase-contrast images). Scale, 100 μm **(A–C)** or 50 μm **(D)**. **(A)** HDMECs (initial seeding); **(B)** fibroblasts (passage number 5); **(C)** keratinocytes (passage number 1); **(D)** melanocytes (passage number 1).

The present protocol allows isolation and culture of HDMECs, fibroblasts, keratinocytes, and melanocytes (illustration of cultured cells: Figure 6). Pitfalls, when they exist, are outlined directly in the protocol described above. The major advantage is to obtain patients' cells which can be either grown, either analyzed with single-cell transcriptomics. However, the latter is not possible on freshly isolated cells for epidermal cells, as the present technique implies that they are separated later on.

**Figure 7:**
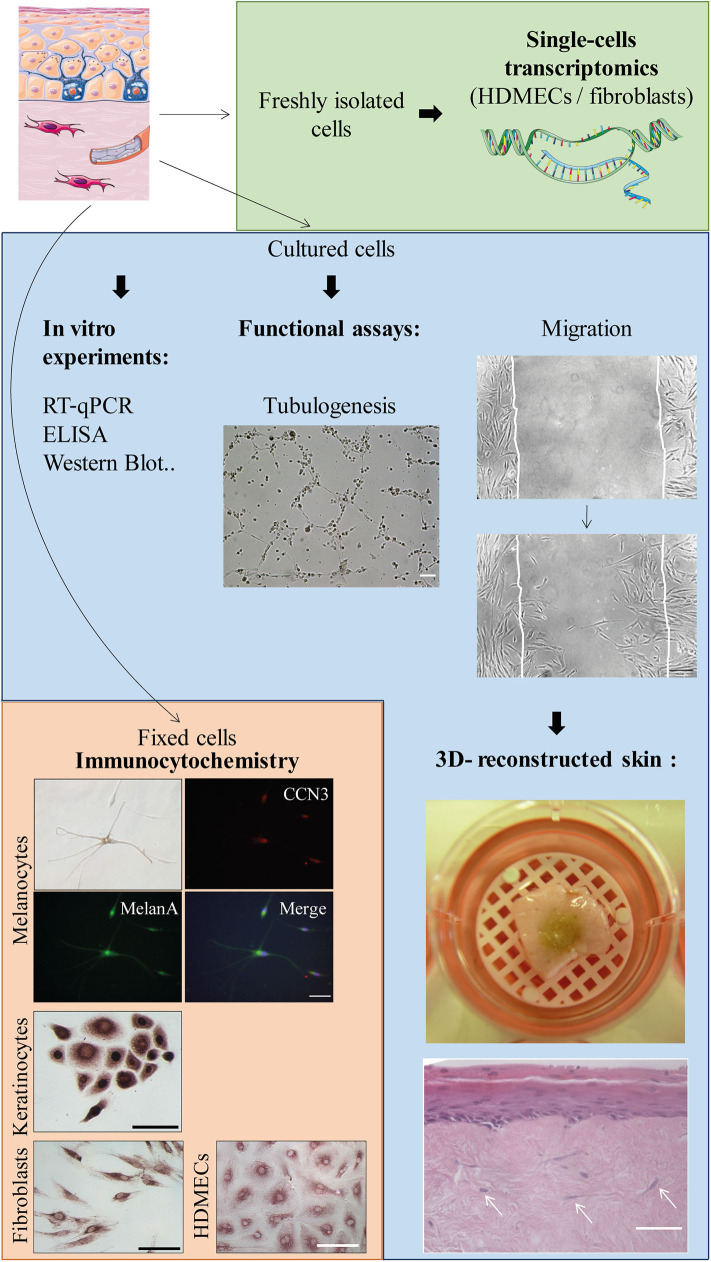
Possible applications of the protocol (non-exhaustive list). Scale bars, 100 μm unless notified otherwise. From upper to lower panels: Tubulogenesis assay on Matrigel™ (or *in vitro* angiogenesis assay): picture taken 6 h after the beginning of the experiment with SSc HDMECs. Migration (or scratch assay) with fibroblasts: second picture taken 22 h after the beginning of the experiment. Skin equivalent (reconstructed epidermis): picture shows the macroscopical aspect of a pigmented reconstructed epidermis on a matrix of dead de-epithelialized dermis (DDD) implemented with fibroblasts. Histological image shows Hematoxylin-Eosin staining of the reconstructed skin with a multilayered differentiated epidermis and dermal fibroblasts within the DDD matrix (white arrows). Scale bar, 20 μm. Immunocytochemistry: experiments were performed to evidence CCN3, an ubiquitous multimodular protein implicated in melanocyte adhesion to the basal epidermal layer as well as angiogenesis [Ricard et al. *Experimental Dermatology* 2012 (6)—Henrot et al. *Journal of Investigative Dermatology* 2020 (7)]. Melanocytes (upper panel): double immunocytochemistry staining using antibodies against CCN3 and MelanA, which is a specific cytoplasmic marker. Nuclei are stained in blue with DAPI. Keratinocytes, fibroblasts, and HDMECs: immunostaining with anti-CCN3 antibody and revelation with horseradish-peroxydase and ImmPACT VIP™ as a substrate.

Concerning HDMECs, the proposed technique (mechanical extraction) allows efficient purity of HMECs culture, with an overall success rate of around 80 % (from our personal experience) as opposed to the conventional technique (dermal digestion with collagenase) (5), which gives pure and sustained HDMECs culture in about 30 % of the cases (also personal experience) (Figure 4). Cells obtained with our technique are in sufficient number for Western Blot or RT-qPCR experiments, as well as functional assays such as migration or *in vitro* angiogenesis assays (Figure 7). Also from personal experience, mechanical extraction of HDMECs directly gives a pure HDMECs culture in around 80% of the cases—for the 20% remaining cases, additional purification methods such as differential trypsinization and/or immunomagnetic cell sorting have to be used in order to obtain a pure culture. Directly obtaining a pure HDMECs culture is of major interest in order to use cells at early passages for subsequent experiments. From our personal experience, HDMECs should not be used beyond the 6th passage.

Fibroblast culture is relatively easy and the present protocol allows the isolation and culture of SSc fibroblasts with a success rate close to 100% from our personal experience. Cells are also in sufficient number for Western Blot, RT-qPCR, or functional assays. From our personal experience, fibroblasts should not be used beyond the 6th or 7th passage.

Both HDMECs and fibroblasts can be kept frozen and grow back correctly once thawed.

Keratinocytes can be obtained from most samples, with a success rate of about 90% from our personal experience. However, cells should be used immediately as they do not grow well after freezing. Best results are also obtained if cells are used within the second passage, in order to avoid keratinocytes differentiation. Keratinocytes differentiation is easy to spot as cells become visibly bigger and stop growing (Supplementary Figure 1).

Melanocytes can also be grown from most samples, with a success rate of around 60% (personal experience). Of note, difficulties to obtain SSc melanocytes are also applicable to healthy skin, particularly from small skin samples as melanocytes represent around 5% of epidermal cells. Cells grow in sufficient number for immunocytochemistry or ELISA experiments. However, given the small sample size, it is quite difficult to obtain enough cells to perform Western Blot or RT-qPCR experiments. SSc melanocytes also do not grow back very well after freezing (which is also true for melanocytes grown from healthy skin). For detailed explanations concerning melanocytes culture in general, see (8).

Of note, as showed in Figure 1, trypsin usually allows separating the epidermis in two parts: the basal layer, constituted by melanocytes and keratinocytes stem cells. When only epidermal cells are required, conventional protocols use skin digestion with trypsin in order to seed only the cells from the basal layer. However, in the current protocol, a digestion with dispase is needed in order to separate the epidermal sheet from the dermis. Therefore, trypsin is used here in order to help digesting inter-cellular adhesions between epidermal cells. Some of the cells that are seeded afterwards may not be stem cells as they originate from upper layers of the epidermis, but this should not be a limitation as they will quickly be overgrown by keratinocytes stem cells.

### Applications

All cells grown from the biopsy are suitable for immunocytochemistry, whether fluorescent or using enzyme-labeled detection system. Cultured cells can also be used for functional assays such as migration (scratch assays) or *in vitro* angiogenesis assays. Finally, cultured cells can be implemented in 3D-skin models (or skin equivalents) (Figure 7). For example, our lab has a long-lasting experience of reconstructed epidermis on a matrix of dead de-epithelialized dermis. Such model allows the formation of a multi-layered epidermis containing both keratinocytes and melanocytes (seeded at a respective proportion of 95/5%). The underlying matrix can also be implemented with dermal cells (fibroblasts and HDMECs) in order to expose the epidermis to secretions from dermal cells (Figure 7 and Supplementary Figure 2). Such model would highly benefit from SSc cells, in order to reproduce the complex interactions between dermal and epidermal cells.

## Discussion

Isolating primary skin cells in SSc can be a struggle due to small sample size as well as skin condition. Here, we present a protocol allowing the isolation of the four main types of skin cells: HDMECs, fibroblasts, keratinocytes, and melanocytes, from a single 4 mm-punch biopsy. Dermal cells (fibroblasts and HDMECs) can be immediately used after skin dissociation, notably for molecular biology experiments such as single-cell transcriptomics; they can also be used for functional assays or 3D-skin models after a few passages. Epidermal cells (keratinocytes and melanocytes) are initially cultured together and can be separated at the first passage in order to be used for functional assays.

Fibroblasts are relatively easy to culture and many protocols describe their isolation (9). However, culturing HDMECs is a more delicate issue. Most published protocols use foreskin tissue, which gives highly proliferative HDMECs culture but poorly reflects adult skin characteristics (10). Yet, HDMECs are cells of major importance concerning SSc pathophysiology: it is currently discussed whether they are key cells initiating fibrotic events, as vascular manifestations such as Raynaud's phenomenon often precede the development of the disease (11). Therefore, having access to patients' cells is a crucial challenge. A well-known problem for HDMECs culture is contamination by surrounding fibroblasts, an issue further enhanced in SSc skin where activated fibroblasts bear extensive proliferative capacity over HDMECs (12). A mechanical extraction of HDMECs possibly combined to immunomagnetic cell sorting, as proposed in the current technique, ultimately allows better specificity of HDMECs culture. If the experimentator is properly trained for mechanical extraction, the immunomagnetic cell sorting can be dispensable; the extracted HDMECs population can be immediately used with a high purity ratio if single-cell experiments are planned. Avoiding immunomagnetic cell sorting could also be useful due to the potential influence of anti-CD31 antibodies coupled to microbeads on HDMECs function (13). Other protocols also describe isolation of HDMECs, such as a protocol using enzymatic digestion and the perfusion technique, specifically designed for SSc skin (14); however, this protocol does not allow culture of epidermal cells. More recently, another protocol has been published allowing culturing of HDMECs and also using mechanical extraction (15); however, this protocol was not specifically optimized for SSc cells, did not allow epidermal cell culture, and requires bigger pieces of skin than a punch biopsy.

Several protocols also well describe epidermal cells culture, such as the paper from Tsuji and Karasek back in 1983, describing melanocytes isolation from newborn skin (16), or the more recent protocol from Wang et al. (17). However in the latter, minimal required biopsy size was 6 × 3-mm punch biopsies (e.g., 42 mm2), which is bigger than the current proposed biopsy size. Morevoer, to our knowledge, no report specifically concerns SSc keratinocytes and melanocytes.

Finally, to our knowledge, only one report describes a protocol aiming to culture keratinocytes, fibroblasts and HDMECs from a single punch biopsy (18). This method already included the mechanical extraction of HDMECs. However, the protocol was not optimized for SSc cells (in particular, HDMECs culture was not detailed) and the possibility to culture melanocytes was not addressed. Altogether, we believe that the current protocol is the only one allowing isolating the four main types of skin cells from a small biopsy in SSc patients.

Epidermal cells, namely keratinocytes and melanocytes, are initially cultured together. Indeed, this step is essential to favor melanocytes growth, which is almost impossible without keratinocytes. However, they are easily separated after a few days, also allowing single-cell experiments, even if such experiments would not be performed directly on freshly isolated cells. After this first passage, keratinocytes and melanocytes are cultivated separately. As melanocytes do not proliferate highly on their own, a pure melanocytes culture could be mostly used to perform immunocytochemistry experiments. However, they can also be used in co-culture experiments (for example with keratinocytes), using a controlled combination of healthy and SSc cells.

Of course, skin cells are prone to lose their original phenotype during successive passages. This is particularly true for keratinocytes, which differentiate rapidly in culture. For these reasons, experimentators are advised to use cells within a few passages, and to favor experiments performed immediately after cell extraction whenever possible. We also recommend to check the morphology of cultured cells and to monitor their growth rate in order to ensure the viability of the culture.

Skin 3D models, or skin equivalents, are a powerful tool in translational research, allowing to test the effects of drugs or to modelize the cellular cross-talk in complex diseases such as SSc (19). This kind of model can highly benefit from primary cells incorporation, thus better reflecting cellular interactions, and even more if primary cells come from patients. Our team has a long-lasting experience in 3D skin models such as reconstructed multi-layered epidermis on a matrix of dead de-epithelialized dermis (DDD) (20). This kind of model, initially aimed at studying epidermal homeostasis, has then been implemented with dermal cells such as fibroblasts and HDMECs (Figure 7 and Supplementary Figure 2). Other 3D models could benefit from patient's cells. Recently, an elegant paper reported a skin equivalent using primary HDMECs forming a functionally vascular network, fibroblasts, and keratinocytes (21). In this model, fibrosis was induced with TGF-β exposure. Thus, such model could highly benefit from the implementation of patients' cells, as well as the addition of melanocytes. Another very interesting report showed the generation of a skin organoid using SSc patients induced pluripotent stem cells (iPSCs), which had been differentiated into fibroblasts and keratinocytes (22). However, iPSCs culture is very delicate and costly, and do not allow the complete control of the differentiation state. Altogether, our protocol provides a quick and efficient way to obtain a primary culture of the four main types of skin cells, allowing their use in skin equivalents.

Our protocol has been adapted to fit the particularities of SSc cells, but works quite efficiently with healthy skin, from our personal experience. Moreover, although we have not tried it directly, it could also be used with any other skin disease. Indeed, many skin diseases rely on abnormalities of several skin cells, such as vitiligo where both epidermal and dermal cells are affected (23). Moreover, dermal digestion with collagenase also allows isolating other cells than HDMECs and fibroblasts. Theoretically, immune cells such dendritic cells or cutaneous T lymphocytes could further be extracted from the dermis in order to be used for characterization or functional experiments, for example by combining our method to other published protocols (24, 25). Such additional methods should not alter the performance of our protocol concerning HDMECs culture (as it represents the first step of dermal digestion) nor fibroblasts culture (as this part is relatively easy). However, the yield of immune cells extraction is probably more hazardous and will possibly require additional digestion enzymes. Ultimately, the current protocol could be adapted to other tissues, by dissociating and cultivating separately epithelial and connective tissue cells.

To conclude, performing experiments on primary cells is essential in the field of translational research, especially for diseases whom pathophysiology is complex and where different cell types may be simultaneously affected. Cells isolated from patients' samples can be further used in 3D skin models, in order to better reproduce the disease complex pathophysiology.

## Data Availability Statement

The original contributions presented in the study are included in the article/Supplementary Material, further inquiries can be directed to the corresponding author/s.

## Ethics Statement

The studies involving human participants were reviewed and approved by the appropriate institutional ethical committee (CPP, 2012 A00081-42, Aquitaine). The patients/participants provided their written informed consent to participate in this study.

## Author Contributions

PH, MC, CP, PL, M-ET, and EL contributed to the conception of the protocol. PH, PL, MC, DL, and EL contributed to data acquisition. PH wrote the first draft of the manuscript. All authors contributed to manuscript revision, read, and approved the submitted version.

## Conflict of Interest

The authors declare that the research was conducted in the absence of any commercial or financial relationships that could be construed as a potential conflict of interest.

## Supplementary Material

Supplementary Figure 1 HDMECs passaging.

Supplementary Figure 2 differentiated keratinocytes.

Supplementary Figure 3 skin equivalent with HDMECs.

Supplementary Video 1 epidermal sheet separation.

Supplementary Video 2 endothelial cells mechanical extraction. Text: protocol for CD31 and CD45 labeling before FACS analysis and immunomagnetic cell sorting.
