# Supplementary material for: A Method for Isolating and Culturing Skin Cells: Application to Endothelial Cells, Fibroblasts, Keratinocytes, and Melanocytes From Punch Biopsies in Systemic Sclerosis Skin
Source: Front Immunol. 2020 Oct 7;11:566607. doi: 10.3389/fimmu.2020.566607 (PMC7575752; doi:10.3389/fimmu.2020.566607)
Supplement: Supplementary Figure 1 — HDMECs passaging. [file Table_1.docx]

Supplementary Material

# Supplementary Data

**Protocol for CD31 labeling before FACS analysis:**

- Aspirate and discard the supernatant of cultured cells, rinse the flask with HBSS, add 0,5 mL of TE 10%.

- Incubate at 37°C for 3-5 minutes, regularly check under the microscope if cells are detached; gently tap the flask if necessary.

- When cells are detached, block TE action with FCS 10%. Collect the cell suspension and count cells. Centrifuge 5 minutes at 1500 rpm. Discard the supernatant.

- Experiment should be performed with a minimum of 10 000 cells. Incubate the cell pellet with 3 to 5 µL of fluorescence-labeled anti-CD31 antibody (e.g., APC-conjugated anti-CD31 antibody, clone AC128, Miltenyi), mix gently (a quick vortex is best), then add 100 µL of FACS buffer. Incubate for 15 minutes at 4°C away from daylight.

- After incubation, add 5 mL of FACS buffer and centrifuge 5 minutes at 1500 rpm. Discard the supernatant. Resuspend the cell pellet in 200 µL of FACS buffer and store at 4°C away from daylight until FACS analysis.

**Protocol for immunomagnetic cell (HDMECs) sorting (CD31-positive cells selection):**

- Aspirate and discard the supernatant of cultured cells (in this case a mixture of fibroblasts and HDMECs), rinse the flask with HBSS, add 0,5 mL of TE 10%.

- Incubate at 37°C for 3-5 minutes, regularly check under the microscope if cells are detached; gently tap the flask if necessary.

- When cells are detached, block TE action with FCS 10%. Collect the cell suspension and count cells. Centrifuge 5 minutes at 1500 rpm. Discard the supernatant.

- Resuspend the cell pellet with 60 µL of HDMECs culture medium (MV2, Promocell) (alternatively, with FACS buffer). Add 20 µL of FC blocking reagent and mix gently (a quick vortex is best); then add 20 µL of magnetic-labeled anti-CD31 beads (e.g. 130-091-935, Miltenyi) and mix gently by rotating the tube. Incubate for 15 to 20 minutes at 4°C, away from daylight.

- Meanwhile, place a LS column in the separator (e.g. QuadroMACS^TM^ Separator (MACS)) and keep the syringe near, both in a sterile fashion. Equilibrate the column by adding minimum 3*1 mL of HDMECs culture medium (MV2). Do not forget to place a tube underneath the column (labeled “CD31-“). For a cheaper alternative, use FACS buffer (in that case, place a “trash”-labeled tube underneath the column). This step is quite long, so it should be performed during magnetic beads incubation.

- After incubation, add 1 mL of MV2 (alternatively, FACS buffer) in the tube and centrifuge 5 minutes at 1500 rpm. Discard the supernatant.

- Resuspend the cell pellet in 1 mL of MV2 and gently pour it into the column (check that the underneath collecting tube is well placed). For best results, avoid bubbles during the whole procedure.

- Rinse the column 3 times with 1 mL of MV2 (each time, you can pour 1 mL of MV2 into the tube where HDMECs were incubated with magnetic beads, in order to retrieve as many cells as possible).

- (quick gesture is advised at this stage) Take the column from the separator, place it on a clean 50 mL-tube named “CD31+” (for LS-column; otherwise, for MS-column you’ll have to use a 15 mL-tube). Add 5 mL of MV2 into the column and push through using the syringe. The column itself should not be rinced and can be discarded afterwards. Collect a small fraction for cell counting and if desired, the rest of the cells can be directly seeded afterwards. Alternatively, they can be immediately used for experiments, but as the procedure “disturbs” them a little bit, it is best to let them rest before performing any experiment.

- Count the CD31-negative fraction (which are fibroblasts), centrifuge 5 minutes at 1500 rpm. Discard the supernatant and resuspend the cell pellet in the appropriate culture medium (i.e., DMEM + 10% FCS + 1% PS). Seed at the appropriate concentration.

- Last, calculate the yield of the immunomagnetic cell sorting by adding HDMECs + fibroblasts number and dividing it by the initial cell number.

**Protocol for immunomagnetic cell (HDMECs) sorting (CD45-positive cells depletion):**

Follow the same steps as described above, except for:

- the column, which should be an LD column;

- at the end of the isolation, the targeted cells (CD31^+^CD45^-^) are in the flow-through fraction, which should be centrifugated before being resuspended and seeded in the appropriate culture medium.

**Supplementary video 1**: epidermal sheet separation (step 2 from section 3.2).

**Supplementary video 2**: endothelial cells mechanical extraction (step 4 from section 3.2).

# Supplementary Figures and Tables

## Supplementary Figures

## Supplementary Figure 1: HDMECs passaging. Scale bar, 100 µm.

The first passage for HDMECs can be performed when cells islets have reached sufficient size (illustration: SSc HDMECs, passage 0), even if the well is not confluent.


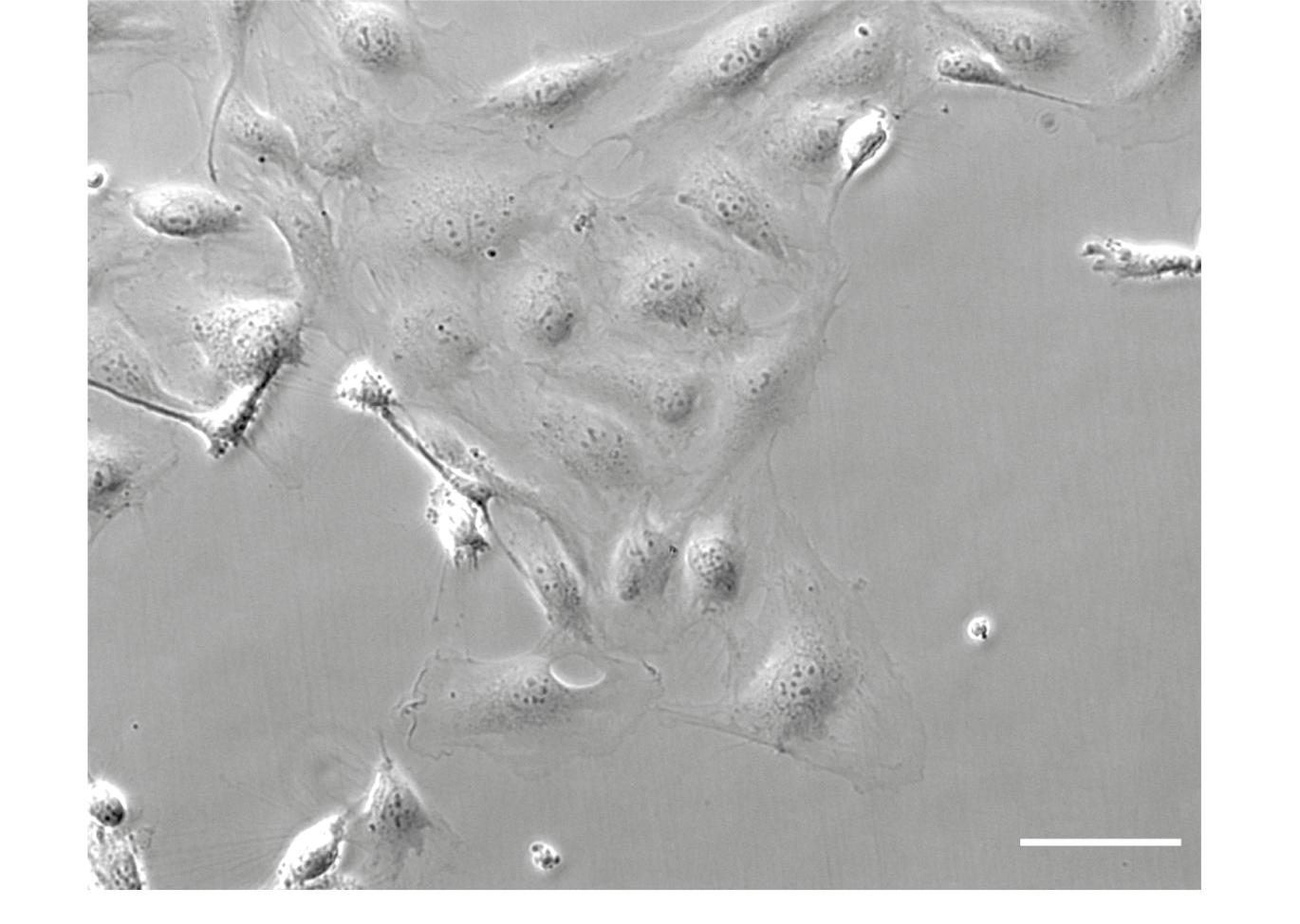


**Supplementary Figure 2:** **differentiated keratinocytes**. Scale bar, 100 µm.

Differentiated keratinocytes (black arrow) are not suitable for cell culture experiments. They are easy to spot as their size is clearly bigger than other keratinocytes. Presence of differentiated cells can be avoided if keratinocytes are used before the second passage and are passaged when they reach maximum 75% of confluence.

**
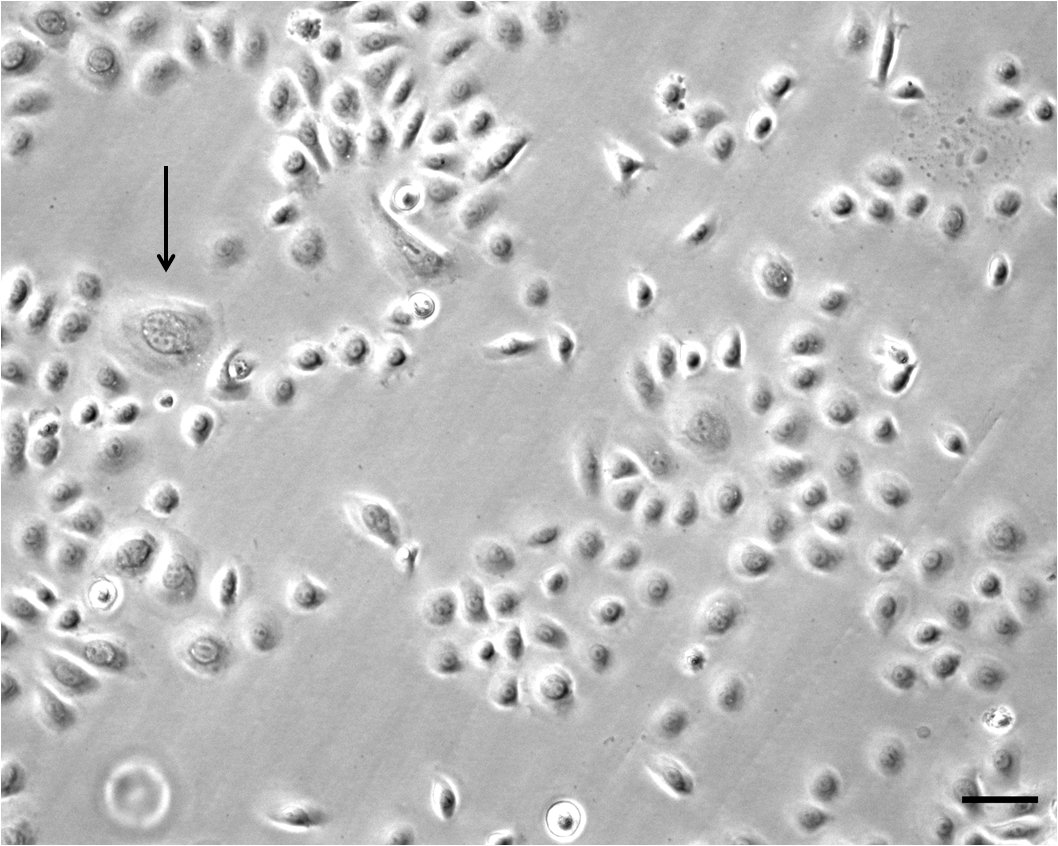
**

**Supplementary Figure 3: skin equivalent with HDMECs.** Scale bar, 20 µm.

Other example of skin equivalent, where the matrix is an artificially made acellular dermal template of collagen-glycosaminoglycan (Integra™), implemented with HDMECs. Immunostaining using an anti-CD31 antibody (revelation with horse-radish peroxidase using Novared™ as a substrate) shows HDMECs organized in a round shape evoking a dermal vessel (black arrow).

Courtesy of Mrs Vanessa Bergeron.


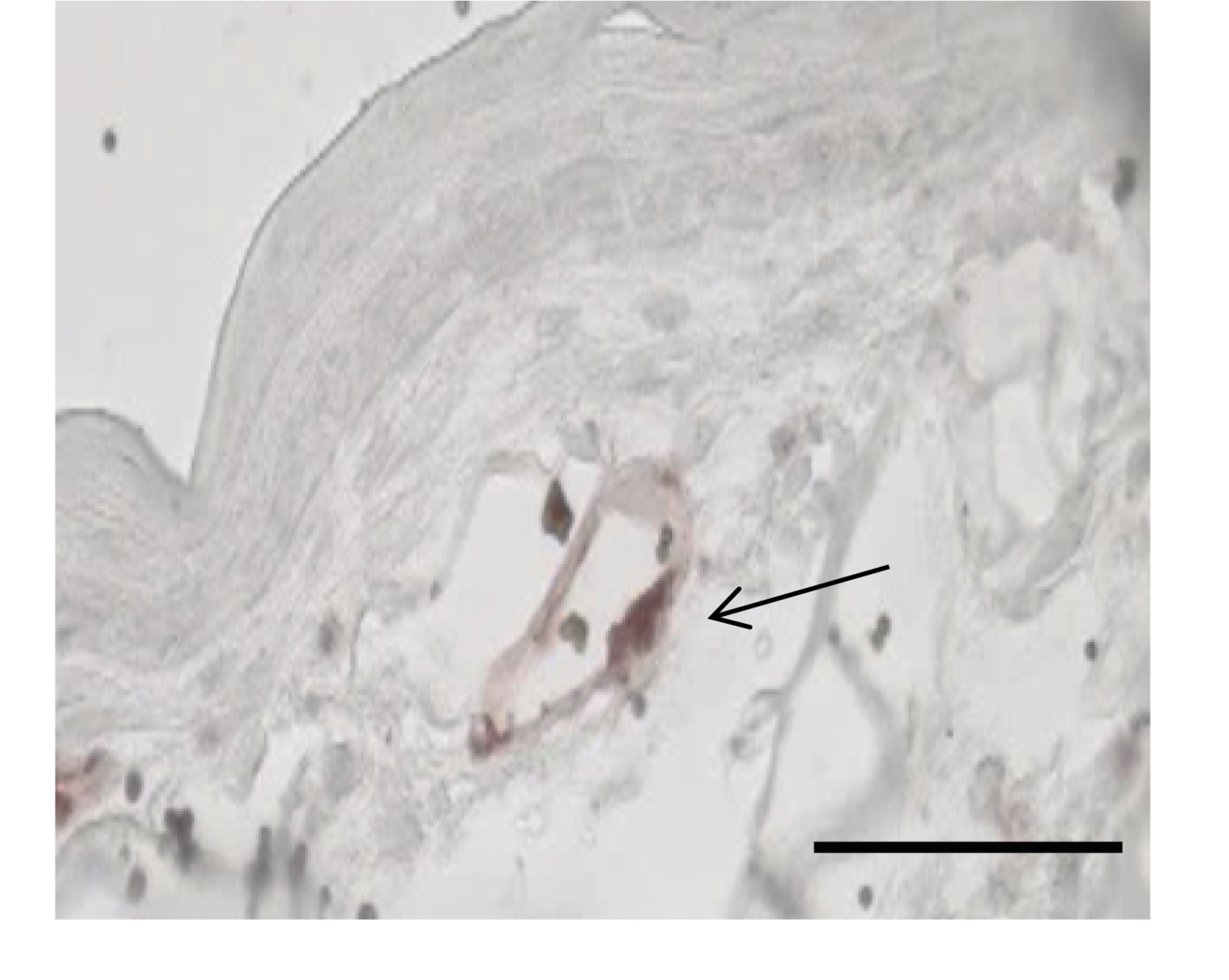


**
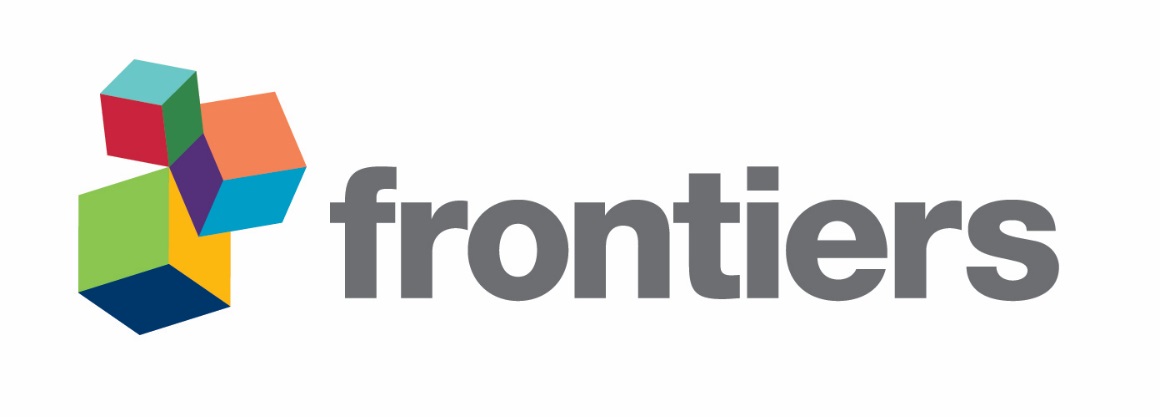
**
